# Concentration of major vitamins in critically ill patients

**DOI:** 10.1186/cc10759

**Published:** 2012-03-20

**Authors:** H Hayami, K Mizutani, M Shiota, N Nakayasu, T Masubuchi, M Idei, T Gotoh

**Affiliations:** 1 Yokohama City University Hospital, Yokohama, Japan

## Introduction

Commercially available vitamin solutions have been improved during the last decade, but there have been a few recent reports on deficiency occurring in the ICU setting. In general, daily delivery of a comprehensive modern vitamin regimen will suffice and TPN solution which contains vitamins and trace elements is now widely used because of the view of convenience and infection control. But the dose of vitamins is determined by the American Medical Association (AMA) recommendation which is based on requirement by healthy subjects and it is not clear whether the dose is applicable for critically ill patients. We measured the concentration of major vitamins in 19 critically ill patients who stayed in the ICU and analyzed those treated for more than 3 weeks.

## Methods

Of 19 patients, 10 were treated for more than 3 weeks under artificial ventilation; seven of which received renal replacement therapy (RRT). Early enteral nutrition was established in six patients who were assessed to have normal intestinal function. For those patients who were diagnosed to have malfunction in intestine, nutrition was supplied via peripheral route for 1 week, and led to total parenteral nutrition after 1 week. Multivitamin product (Vit B1 3 mg, Vit B6 4 mg, Vit C 100 mg, Folate 400 mg, and so on) was administered from day 0. We measured the concentration of those vitamins every 7 days.

## Results

Concentrations of Vit B1, Vit 12, and Folate were 37 ± 16 pg/ml, 1,068 ± 1,702 pg/ml, and 9.9 ± 14.4 ng/ml on day 7, and there was tendency of increasing to normal range subsequently. On the other hand, the concentration of Vit C was low (2.5 ± 2.4 g/ml: median 1.75) on day 7, and it remained low through 3 weeks (median 2.0 g/ml on day 21). Especially, the concentration of Vit C was extremely low in seven patients who received RRT (median 1.0 g/ml) on day 7 compared with those without RRT (median 2.6 g/ml, *P *= 0.05). We administered a high dose of Vit C (ascorbic acid 1,000 mg/day) for three patients in this group but restoration to normal range was seen in only two patients.

## Conclusion

In critically ill patients, especially those who received RRT, the concentration of water-soluble vitamins such as Vit C remained low even when they received the AMA recommended dose. Measurement of vitamins and additional administration will be needed as necessary depending on the disease condition.

